# Mutational spectrum for guiding the decision of adjuvant treatment in patients with resected biliary tract carcinoma

**DOI:** 10.1002/cam4.6261

**Published:** 2023-06-21

**Authors:** Yunfeng Li, Chaochao Tan, Xinmin Yin, Siwei Zhu, Rongyao Cai, Chunhong Liao, Yifei Wu, Qihong Zeng, Chengzhi Cai, Wang Xie, Xiangyu He, Hao‐quan Wen, Guomin Lin, Qingqing He, Tingting He, Peng Gu, Chang‐jun Liu

**Affiliations:** ^1^ Department of Hepatobiliary Surgery Hunan Provincial People's Hospital, The First Affiliated Hospital of Hunan Normal University Changsha China; ^2^ Department of Clinical Medical Laboratory Hunan Provincial People's Hospital, The First Affiliated Hospital of Hunan Normal University Changsha China; ^3^ Shanghai OrigiMed Co., Ltd. Shanghai China

**Keywords:** adjuvant therapy, bile duct cancer, cholangiocarcinoma, gallbladder cancer, next‐generation sequencing

## Abstract

**Background:**

Systemic chemotherapy or chemoradiation therapy has proven to be effective in treating advanced biliary tract carcinoma (BTC). However, its efficacy in the adjuvant setting remains controversial. Therefore, this study aimed to determine the prognostic significance of genomic biomarkers in resected BTC and their potential role in stratifying patients for adjuvant treatment.

**Methods:**

We retrospectively reviewed 113 BTC patients who underwent curative‐intent surgery and had available tumor sequencing data. Disease‐free survival (DFS) was the primary outcome examined and univariate analysis was used to identify gene mutations with prognostic value. Favorable and unfavoratble gene subsets were distinguished from the selected genes through grouping, respectively. Multivariate Cox regression was used to identify independent prognostic factors of DFS.

**Results:**

Our results indicated that mutations in *ACVR1B*, *AR*, *CTNNB1*, *ERBB3*, and *LRP2* were favorable mutations, while mutations in *ARID1A*, *CDKN2A*, *FGFR2*, *NF1*, *NF2*, *PBRM1*, *PIK3CA*, and *TGFBR1* were unfavorable mutations. In addition to age, sex, and node positive, favorable genes (HR = 0.15, 95% CI = 0.04–0.48, *p* = 0.001) and unfavorable genes (HR = 2.86, 95% CI = 1.51–5.29, *p* = 0.001) were identified as independent prognostic factors for DFS. Out of the 113 patients, only 35 received adjuvant treatment whereas the majority (78) did not. For patients with both favorable and unfavorable mutations undetected, adjuvant treatment showed negative effect on DFS (median DFS: S441 vs. 956 days, *p* = 0.010), but there was no significant difference in DFS among those in other mutational subgroups.

**Conclusions:**

Genomic testing might be useful in guiding the decisions regarding adjuvant treatment in BTC.

## 1 INTRODUCTION

Biliary tract carcinoma (BTC), which mainly includes gallbladder cancer and cholangiocarcinoma, is characterized by late‐stage diagnosis, a poor prognosis, and low survival rate.^1^, ^2^, ^3^ The pathogenesis of BTC is complex and not yet fully understood. In 2018, the incidence and mortality rates of BTC were 4.12 and 3.25 per 100 thousand people in China, respectively.^4^ Despite the low incidence rate of BTC, the disease remains a significant burden in China due to the country's large population.

Currently, surgery is the only curative approach for treating BTC. However, only a quarter of BTC patients are eligible for surgery, and the postoperative recurrence rates are high.^5^ Several clinical trials have investigated the use of adjuvant chemotherapy to improve the prognosis in BTC patients, but most have been unsuccessful. The initial phase III randomized trial aimed to evaluate adjuvant chemotherapy with fluorouracil and mitomycin C or mycin C in located patients (139 with CCA and 140 with gallbladder cancer included). This trial revealed a significantly improved 5‐year disease‐free survival (DFS) for patients with gallbladder cancer undergoing chemotherapy (20.3% vs. 11.6%, *p* = 0.021), which supporting the notion that adjuvant chemotherapy may offer survival benefits for patients with gallbladder cancer who are undergoing resection.^6^ The BILCAP study,^7^, ^8^ which compared adjuvant capecitabine treatment with observation only in BTC patients, was another successful trial in postoperative adjuvant chemotherapy. Patients who received adjuvant capecitabine had both a longer recurrence‐free survival (RFS) (25.9 vs. 17.6 months, hazard ratio (HR) = 0.71) and overall survival (OS) (52.7 vs. 36.1 months, HR = 0.75 in the per‐protocol population) compared to those in the observation arm. Based on this study, clinical guidelines from organizations such as ASCO and CSCO recommend capecitabine as the standard adjuvant chemotherapy regimen for BTC.^9^, ^10^ Other major trials, such as the PRODIGE 12‐ACCORD 18 study, using a gemcitabine‐oxaliplatin combination regimen,^11^ or the ASCOT study using an S‐1 regimen,^12^ failed to show significant improvement in prognosis or are still ongoing. In the past decade, immune checkpoint inhibitor (ICI) therapies have been one of the major breakthroughs in the clinical treatment of cancer. Clinical studies have shown that second or late‐line ICIs monotherapy can prolong the medium OS of advanced BTC patients, with a range from 5.2 to 14.2 months.^13^ However, the field of postoperative adjuvant immunotherapy for BTC has been scarcely explored. Given the clinical and pathological heterogeneity of BTC and its inadequate response to systemic therapies, biomarkers may be necessary to identify appropriate patients for adjuvant treatment.

The genomic landscape of BTC has been extensively studied.^14^, ^15^ However, only a few studies have explored the prognostic value of genomic biomarkers in BTC. Conci et al^16^ reported that *TP53* and *ARID1A* mutations were associated with a higher risk of recurrence after surgery. Churi et al^17^ suggested that *TP53*, *KRAS*, and *BAP1* mutations predicted dismal prognosis in advanced BTC patients, unlike mutations in FGF pathway that predicted a prolonged prognosis. In intrahepatic cholangiocarcinoma, *TP53*, *KRAS*, and *CDKN2A* mutations were found to cause poor prognosis, but *IDH* mutated patients had a favorable prognosis.^18^, ^19^ Regardless of treatment, Jolissaint et al^20^ stated that node‐positive intrahepatic cholangiocarcinoma patients had worse OS when *TP53*, *KRAS* or *CDKN2A/B* mutations were detected. Intriguingly, a study recruiting advanced BTC patients receiving platinum‐based chemotherapy found that mutations of the DNA damage repair (DDR) pathway, which occurred in 63.5% of patients, predicted a better prognosis progression free survival (PFS) (6.9 vs. 5.7 months, *p* = 0.013; OS: 21.0 vs. 13.3 months, *p* = 0.009).^21^ Although these studies disclosed the prognostic significance of genomic biomarkers in BTC, no study had interrogated its clinical value in guiding adjuvant treatment decision. Therefore, in this study, we aimed to examine the possibility of utilizing genomic biomarkers in stratifying surgically‐resected BTC patients.

## 2 MATERIALS AND METHODS

### 2.1 Study design

A total of 113 patients with BTC who underwent curative‐intent surgery between April 2016 and July 2021 at Hunan Provincial People's Hospital were reviewed. The inclusion criteria were as follows: (1) aged 18 years or older; (2) pathologically diagnosed with BTC, which included cholangiocarcinoma and gallbladder cancer; (3) clinical stage I–III and underwent curative‐intent surgery; and (4) tumor sequencing data were available for analysis. The exclusion criteria were as follows: (1) mixed hepatobiliary cancer, ampullary cancer, neuroendocrine cancer, or sarcomatoid cancer; (2) R2 resection margin status; and (3) missing clinical and/or pathological information required for analysis. According to the NCCN guidelines,^22^ the postoperative adjuvant therapy requirements included the following: (1) for patients who have undergone R0 resection for gallbladder or extrahepatic bile duct cancer and have negative regional nodes or carcinoma in situ at the margin, the options of systemic therapy (preferred), clinical trial (preferred), observation alone, or fluoropyrimidine‐based chemoradiation may be considered; and (2) for patients with intrahepatic CCA who have undergone R0 resection, the options of systemic therapy (preferred), clinical trial (preferred), or observation may be considered. The Ethics Committee of the Hunan Provincial People's Hospital approved this study (Approval No.: Research 2022‐53), and informed consent was obtained from all enrolled patients.

### 2.2 Data collection

Clinical data, including information on the patients' age, sex, cancer type, resection margin status, pathological T and N staging, tumor differentiation, microvascular and perineural invasion status, and adjuvant treatment regimens, were collected through medical record. The most commonly used adjuvant treatment regimens were S‐1 based, platinum‐based, ICI‐based, and capecitabine (see Table S1 for details). On average, patients underwent adjuvant treatment was 180 days (with a range of 30–625 days). Follow‐up was conducted in accordance with CSCO guideline.^10^

### 2.3 Sequencing data

Tumor tissue specimens obtained from surgery were processed into formalin‐fixed paraffin‐embedded (FFPE) samples and were collected. All the FFPE samples were reviewed by an independent pathologist to ensure the percentage of tumor cells was sufficient for sequencing. The targeted sequencing panel, Yuansu®450, was applied to FFPE samples and the matched peripheral blood samples were used to detect germline mutations. The experiment was conducted in Shanghai OrigiMed Co., Ltd., Shanghai, China, a laboratory certified by the College of American Pathologists (CAP) and the American Clinical Laboratory Improvement Amendments (CLIA). The procedures for DNA extraction, library preparation, sequencing, and bioinformatics analysis were described in detail in a previous study.^23^

In this study, germline or somatic mutations in *ATM*, *ATR*, *BAP1*, and *BRCA2* were classified as DDR pathway mutations, and somatic mutations in *FGF3* and *FGFR2* were classified as fibroblast growth factor (FGF) pathway mutations.

### 2.4 Statistical analysis

The statistical analysis was performed with R software version 4.1.2. Continuous variables were presented as mean ± SD or median (range) and compared by using *t* test or Mann–Whitney *U* test as appropriate. Categorical variables were presented as frequency (percentage) and analyzed using Chi‐square test or Fisher's exact test as appropriate. DFS was defined as the time from surgery to recurrence, death of any cause, or last consultation day. Survival analysis was performed utilizing Kaplan–Meier curves and compared using the log‐rank test. Multivariate Cox regression analysis was conducted to identify independent prognostic factors. A two‐sided *p* < 0.05 was considered statistically significant.

## 3 RESULTS

### 3.1 Clinical characteristics

In the present study, a total of 113 BTC patients were analyzed (see Figure S1), of whom 35 received adjuvant treatment and 78 did not. Patients' demographic and clinical information were listed in Table 1. The baseline characteristics, including age, sex, resection margin status, T staging, N staging, tumor differentiation, microvascular invasion, and perineural invasion were comparable for patients who received adjuvant treatment and those who did not (*p* > 0.05), except for cancer type (*p* < 0.01) and survival condition (*p* = 0.013), with patients who received adjuvant therapy having a better prognosis.

**TABLE 1:** Baseline information of the enrolled patients.

Clinical factors	Received adjuvant treatment (*n* = 35)	Did not received adjuvant treatment (*n* = 78)	*p* value
Age			
≤60	23 (65.7%)	36 (46.2%)	0.07
>60	12 (34.3%)	42 (53.8%)	
Sex			
Female	15 (42.9%)	36 (46.2%)	0.84
Male	20 (57.1%)	42 (53.8%)	
Cancer type			
eCCA	4 (11.4%)	23 (29.5%)	<0.01
GBC	3 (8.6%)	25 (32.1%)	
hCCA	7 (20.0%)	8 (10.3%)	
iCCA	21 (60.0%)	22 (28.2%)	
Resection margin status			
R0	32 (91.4%)	73 (93.6%)	0.70
R1	3 (8.6%)	5 (6.4%)	
T staging			
1–2	16 (45.7%)	41 (52.6%)	0.81
3–4	18 (51.4%)	34 (43.6%)	
Unknown	1 (2.9%)	3 (3.8%)	
N staging			
Negative	22 (62.9%)	63 (80.8%)	0.11
Positive	11 (31.4%)	12 (15.4%)	
Unknown	2 (5.7%)	3 (3.8%)	
Tumor differentiation			
Low	26 (74.3%)	44 (56.4%)	0.13
Moderate‐high	9 (25.7%)	33 (42.3%)	
Unknown	0 (0%)	1 (1.3%)	
Microvascular invasion			
Present	11 (31.4%)	20 (25.6%)	0.65
Absent	24 (68.6%)	58 (74.4%)	
Perineural invasion			
Present	23 (65.7%)	39 (50.0%)	0.15
Absent	12 (34.3%)	39 (50.0%)	
Survival condition			
Alive	23 (65.71%)	33 (42.31%)	0.013
Dead	11 (31.43%)	27 (34.61%)	
Unknown	1 (2.86%)	18 (23.08%)	

Abbreviations: eCCA, extrahepatic cholangiocarcinoma; GBC, gallbladder cancer; hCCA, hilar chonlangiocarcinoma; iCCA, intrahepatic cholangiocarcinoma.

The median follow‐up duration was 788 days (with a range from 6 to 1869 days), moreover, there was no statistical difference in median follow‐up time between patients received adjuvant therapy and those who did not (338 vs. 200 days, *p* = 0.186). At the time of analysis, 55 patients (48.7%) had experienced a recurrence. In the univariate analysis, the use of adjuvant treatment was not found to be associated with DFS (*p* = 0.320). However, N staging (*p* = 0.044), tumor differentiation (*p* = 0.010), and microvascular invasion (*p* = 0.005) were found to be correlated with DFS (as seen in Figure S2). Other clinical factors, such as age, sex, cancer type, resection margin status, T staging, and perineural invasion, were not found to be associated with DFS (*p* > 0.05).

### 3.2 Genomic characteristics

**FIGURE 1:**
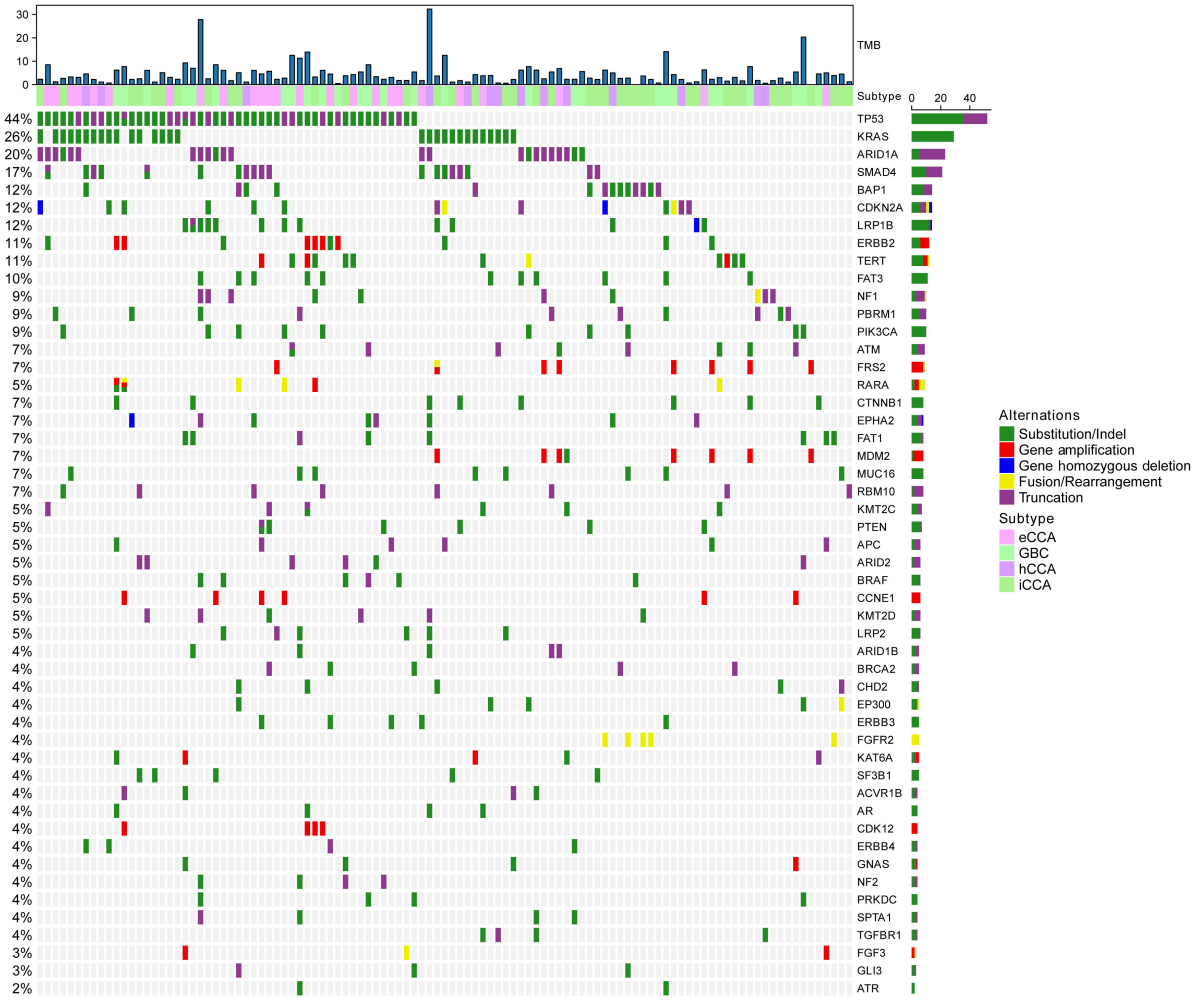
Mutational profiling of the study cohort. eCCA, extrahepatic cholangiocarcinoma; GBC, gallbladder cancer; hCCA, hilar cholangiocarcinoma; iCCA, intrahepatic cholangiocarcinoma; TMB, tumor mutation burden.

The mutational profiling of the enrolled patients was illustrated in Figure 1. The two most frequently mutated genes were *TP53* (*n* = 50, 44%) and *KRAS* (*n* = 29, 26%). Additionally, we detected several mutations that have previously been associated with favorable prognosis, including DDR pathway mutations (*n* = 25, 22%), FGF pathway mutations (*n* = 8, 7%), and *IDH1/2* mutations (*n* = 7, 6%). Interestingly, we observed a significant co‐occurrence between *MDM2* mutation and *FRS2* mutation (*p* < 0.01), while *MDM2* and *TP53* mutations were mutually exclusive (*p* < 0.05; Figure S3). For tumor mutation burden (TMB), the median TMB value was 3.1 mutations/Mb, with eight patients (7%) having a TMB value >10 mutations/Mb and thus considered TMB‐high. Notably, all *FGFR2* fusions were only detected in patients with intrahepatic cholangiocarcinoma, which was expected.

**FIGURE 2:**
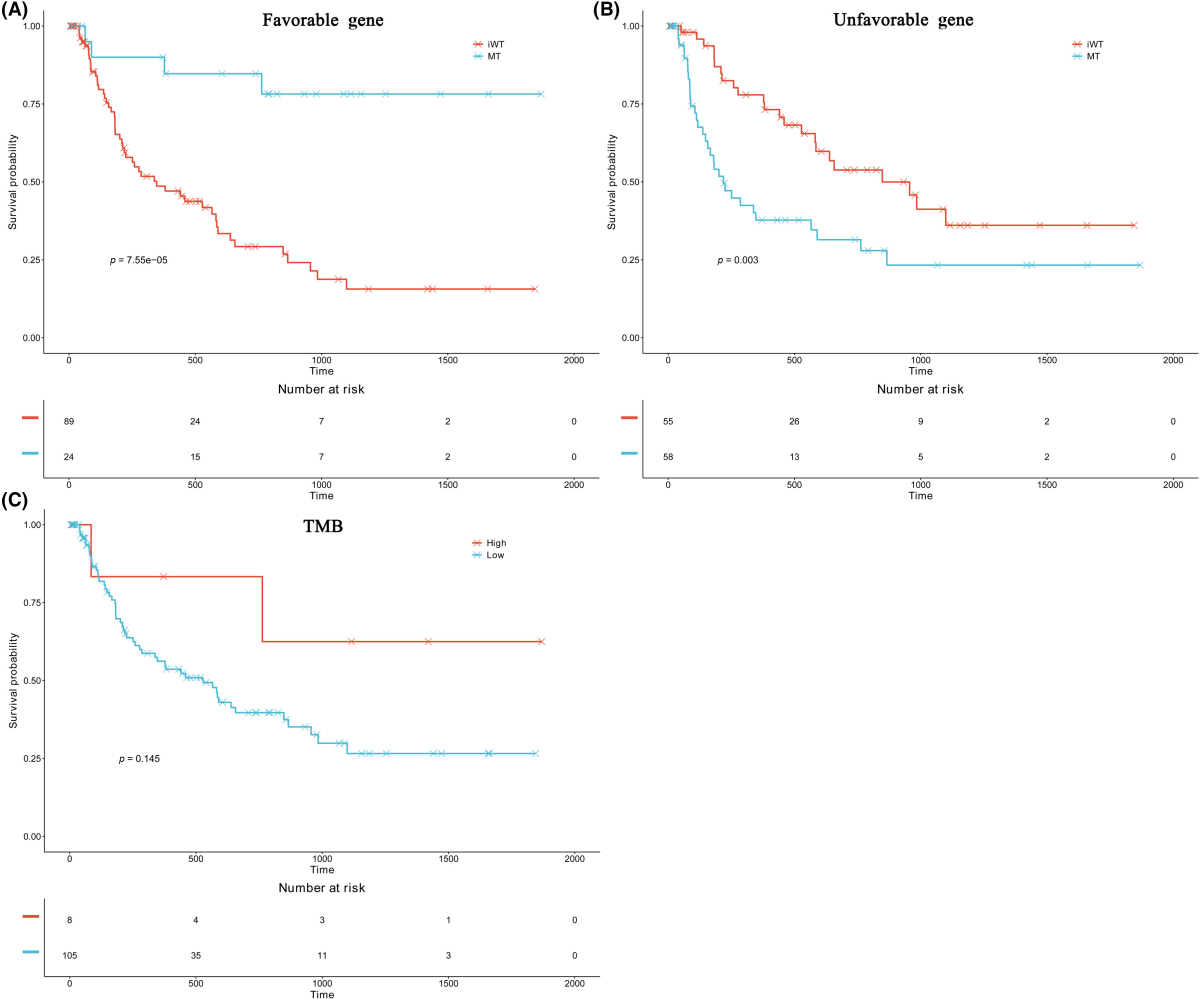
Kaplan–Meier curves according to the genetic factors. Kaplan–Meier curves comparing the DFS of patients (A) with and without mutations in favorable gene subset; (B) with and without mutations in unfavorable gene subset; and (C) TMB low versus high.

In our cohort, we conducted univariate analysis on the top 50 frequently mutated genes (Table S2). As different mutations have diverse effects on DFS, we categorized gene mutations with prognostic value into two subsets: “Favorable genes,” which had a positive effect on DFS (with survival curves of the mutated group above that of the wild‐type group), including mutations in *ACVR1B*, *AR*, *CTNNB1*, *ERBB3*, and *LRP2* (Figure S4A–E); and “Unfavorable genes,” which had a negative effect on DFS (with survival curves of the mutated group below that of the wild‐type group), including mutations in *ARID1A*, *CDKN2A*, *FGFR2*, *NF1*, *NF2*, *PBRM1*, *PIK3CA*, and *TGFBR1* (Figure S5A–H). “Favorable genes” demonstrated a significantly better DFS (median DFS: not reached vs. 347 days, *p* < 0.001; Figure 2A), while “Unfavorable genes” exhibited a significantly worse DFS (median DFS: 219 vs. 848 days, *p* = 0.003; Figure 2B). Furthermore, patients with high TMB demonstrated a trend for better DFS, although this difference was not statistically significant (median DFS: not reached vs. 528 days, *p* = 0.145; Figure 2C).

### 3.3 Multivariate prognostic analysis

We performed a multivariate Cox regression analysis to comprehensively compare the prognostic value of genomic biomarkers and conventional clinical factors. As shown in Table 2, age greater than 60 (HR = 0.37, 95% CI = 0.20–0.71, *p* = 0.003), male gender (HR = 2.03, 95% CI = 1.03–4.02, *p* = 0.041), node positive (HR = 2.77, 95% CI = 1.21–6.34, *p* = 0.016), favorable genes (HR = 0.15, 95% CI = 0.04–0.48, *p* = 0.001), and unfavorable genes (HR = 2.86, 95% CI = 1.51–5.29, *p* = 0.001) were independent prognostic factors for DFS, indicating that mutational spectrum may serve as a strong predictor for prognosis in patients with surgically‐resected BTC.

**TABLE 2:** Multivariate Cox regression for DFS.

Factors	*n*	Hazard ratio (95% CI)	*p* value
Age			
≤60	59	Reference	
>60	54	0.37 (0.20–0.71)	0.003
Sex			
Female	51	Reference	
Male	62	2.03 (1.03–4.02)	0.041
Cancer type			
eCCA	27	Reference	
GBC	28	0.76 (0.23–2.51)	0.657
hCCA	15	0.78 (0.26–2.29)	0.645
iCCA	43	0.93 (0.32–2.71)	0.898
Resection margin status			
R0	105	Reference	
R1	8	1.98 (0.71–5.50)	0.189
T staging			
1–2	57	Reference	
3–4	52	1.23 (0.65–2.32)	0.527
Unknown	4	1.19 (0.18–7.78)	0.859
N staging			
Negative	85	Reference	
Positive	23	2.77 (1.21–6.34)	0.016
Unknown	5	0.84 (0.11–6.18)	0.866
Tumor differentiation			
Low	70	Reference	
Moderate‐High	42	0.57 (0.28–1.16)	0.118
Unknown	1	–	–
Microvascular invasion			
Absent	82	Reference	
Present	31	1.32 (0.62–2.83)	0.472
Perineural invasion			
Absent	51	Reference	
Present	62	1.85 (0.97–3.51)	0.062
Adjuvant treatment			
Did not received	35	Reference	
Received	78	1.39 (0.73–2.68)	0.317
TMB			
High	8	Reference	
Low	105	1.92 (0.36–10.18)	0.442
Favorable genes			
Wild‐type	89	Reference	
Mutated	24	0.15 (0.04–0.48)	0.001
Unfavorable genes			
Wild‐type	55	Reference	
Mutated	58	2.83 (1.51–5.29)	0.001

Abbreviations: eCCA, extrahepatic cholangiocarcinoma; GBC, gallbladder cancer; hCCA, hilar cholangiocarcinoma; iCCA, intrahepatic cholangiocarcinoma.

### 3.4 The prognostic significance of mutational subgroup

**FIGURE 3:**
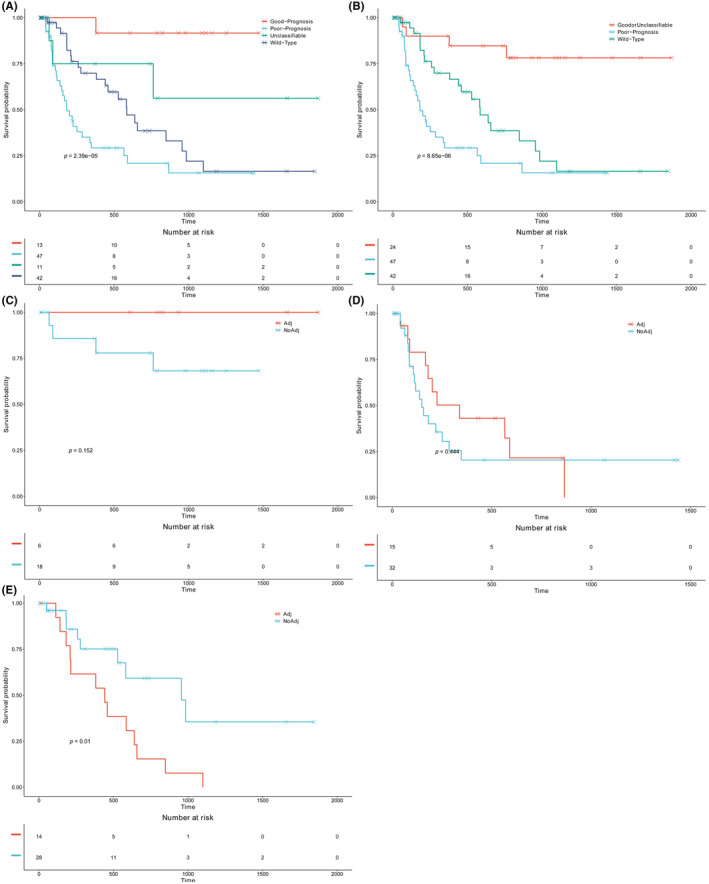
Kaplan–Meier curves according to the mutational subgroups. Kaplan–Meier curves comparing the DFS of (A) Good‐prognosis versus unclassifiable versus wild‐type versus poor‐prognosis subgroups; and (B) good‐prognosis/unclassifiable versus wild‐type versus poor‐prognosis subgroups. Kaplan–Meier curves comparing the DFS of patients who received and did not receive adjuvant treatment in the (C) good‐prognosis/unclassifiable; (D) poor‐prognosis; and (E) wild‐type subgroups.

To better stratify BTC patients based on mutations, we simultaneously considered both favorable and unfavorable genes. We classified the patients into four mutational subgroups: The Good‐Prognosis group, with only favorable gene mutations detected; the Poor‐Prognosis group, with only unfavorable gene mutations detected; the Unclassifiable group, with both favorable and unfavorable gene mutations detected; and the Wild‐Type group, without any favorable or unfavorable gene mutations. Figure 3A displayed the survival curves of these mutational subgroups. The Good‐Prognosis and Unclassifiable groups showed the best prognosis, with a median DFS not reached, and followed by the Wild‐Type group (median DFS 585 days), while the Poor‐Prognosis group had the worst prognosis, with a median DFS of 182 days. Given the small sample size and the comparable prognosis of the Good‐Prognosis and Unclassifiable groups, we opted to merge them into a single group for subsequent analysis (Figure 3B).

### 3.5 The efficacy of adjuvant treatment in each mutational subgroup

To explore the feasibility of utilizing genomic biomarkers to guide the decision‐making process for adjuvant treatment in BTC patients, we conducted a comparison of DFS between patients who received adjuvant treatment and those who did not within each mutational subgroup. Our findings revealed that within the Good‐Prognosis/Unclassifiable group, there was no significant difference in DFS (median DFS not reached, *p* = 0.152; Figure 3C), which suggested that the benefit of adjuvant treatment was somewhat limited. Similarly, in the Poor‐Prognosis group, we observed uncompilable results (median DFS 338 vs. 148 days, *p* = 0.444; Figure 3D). Conversely, patients in the Wild‐Type group who did not receive adjuvant treatment experienced a significantly longer DFS (median DFS 441 vs. 956 days, *p* = 0.010; Figure 3E), implying that adjuvant treatment could potentially be detrimental to this group of patients.

### 3.6 The prevalence of other treatment‐guiding biomarkers in each mutational subgroup

The TMB value could represent a possible biomarkers for predict the efficacy of ICIs.^24^ Among the good‐prognosis/unclassifiable group, wild‐type group, and poor‐prognosis group, there were 4 (17%), 1 (2%), and 3 (6%) patients with high TMB, respectively. Interestingly, among the 5 patients who received adjuvant ICI, the one with the highest TMB value (6.2 mutations/Mb) did not experience recurrence after a 792 days follow‐up. Conversely, all the other 4 patients with relatively lower TMB values (range 1.6–5.1 mutations/Mb) experienced disease recurrence at 141 to 585 days post‐surgery (see Table S1).

## 4 DISCUSSION

There is a lack of evidences supporting the clinical significance of genomic biomarkers in BTC.^16^, ^17^, ^18^, ^19^, ^20^, ^21^ While some studies have examined the efficacy of molecularly guided targeted therapies in the advanced stage setting,^25^, ^26^, ^27^, ^28^ only few studies have investigated the prognostic value of genetic mutations in the resectable cohort.^16^ Currently, according to the various clinical guidelines,^10^, ^22^ the consideration of adjuvant therapy for postoperative BTC patients depends mainly on clinical features such as the successful resection degree and the lymph node metastasis status. As of yet, no study has explored the possibility of guiding adjuvant treatment decisions based on genetic mutations. In agreement with a prior study,^14^ *TP53* and *KRAS* mutations were found to be the most frequently mutated genes in BTC. However, our results differed slightly from past studies^16^, ^17^, ^18^, ^19^, ^20^, ^21^ in univariate analysis. The negative prognostic values of *TP53* and *KRAS* mutations and the positive prognostic values of DDR pathway, FGF pathway, and *IDH1/2* mutations were not statistically significant. These discrepancies could be attributed to the differences in the study population. Previous studies mainly focused on advanced BTCs or a specific subtype of BTC, such as intrahepatic cholangiocarcinoma, whereas our study particularly investigated localized BTC patients who underwent curative‐intent surgery. Indeed, the prognostic value of *ARID1A* and *CDKN2A* mutations in our study was consistent with previous researches.^16^, ^20^ However, when compared to the research that specifically studies surgically resected BTC patients,^16^ our results were partially consistent, as *ARID1A* mutation were demonstrated to have prognostic value, while *TP53* mutation did not. We believe that this inconsistency may be due to differences in race. To the best of our knowledge, our study is the first to explore the comprehensive genomic profile of resectable BTCs using a larger panel (450 vs. 52‐gene), revealing several novel gene mutations with favorable (i.e., *ACVR1B*, *AR*, *CTNNB1*, *ERBB3*, and *LRP2*) and unfavorable (i.e., *ARID1A*, *CDKN2A*, *FGFR2*, *NF1*, *NF2*, *PBRM1*, *PIK3CA*, and *TGFBR1*) prognostic values. In multivariate analysis, N staging was found to be an independent prognostic factor for DFS in surgically resected BTC patients.^29^, ^30^, ^31^ However, both mutational spectrums—“favorable” and “unfavorable” genes can also significantly predict DFS in localized BTC patients, suggesting that mutations were strong prognostic factors along with conventional clinical factors. Combining the two mutation subsets helps in dividing BTC patients into subgroups with significantly different prognosis.

To examine the clinical value of this mutational subgrouping in guiding the decision of adjuvant treatment in BTC, the DFS of the enroll patients who received or did not receive adjuvant treatment in each mutational subgroup was compared. There was no significant difference in DFS in the Good‐Prognosis/Unclassifiable group and the Poor‐Prognosis group, indicating that the benefit of adjuvant treatment was limited. However, strikingly, none of the patients receiving adjuvant treatment in the Good‐Prognosis/Unclassifiable group had disease recurrence during follow‐up, suggesting that adjuvant treatment might be effective in this mutational subgroup. Further study is needed to validate this finding. On the contrary, the DFS was significantly longer for patients who did not receive adjuvant treatment in the Wild‐Type group, implying that adjuvant treatment may be detrimental to patients of this mutational group. The lack of available biomarkers for selecting targeted therapy could be a possible reason for the poor outcome of adjuvant treatment in BTCs. There are a few cases supporting the selection of high TMB patients for ICI in our study, but this required further validation in larger sample sizes.

The current study had several limitations. First, its retrospective nature may introduce selection biases. Second, the small sample size of certain types of patients (such as those with rare mutations or receiving specific adjuvant regimens) greatly limited the reliability of the corresponding subgroup analysis. Third, due to the relatively small number of cases, we combined the pathological subtypes of BTC, including gallbladder and bile duct cancer in our data analysis. However, there may be differences in the biology among these pathological subtypes of BTC. In future studies, we may consider analyzing the data from different pathological subtypes separately. In all, a prospective and systematic study with a larger sample size and specific subgroups is needed to further validate these findings. The importance of genetic biomarkers is increasingly apparent in various types of cancer, particularly in lung and colon cancer. These biomarkers have found application in precision medicine, including auxiliary treatment planning and molecular profiling. As a result, there has been a surge of research focusing on genetic biomarkers associated with clinical therapeutic efficacy. Additionally, there is an urgent need for more clinical research data in the field of biliary tract tumors to further elucidate the importance of genetic biomarkers in precision therapy.

## 5 CONCLUSIONS

To conclude, this study supports the prognostic significance of mutations in localized BTC patients, and the mutational spectrum might guide the decision for adjuvant treatment. The pre‐selection of appropriate patients with genomic biomarkers will be key to the success of upcoming clinical trials studying the efficacy of adjuvant treatment in BTC patients.

## AUTHOR CONTRIBUTIONS

**Yunfeng Li:** Conceptualization (equal); data curation (equal); formal analysis (equal); funding acquisition (lead); writing – original draft (equal); writing – review and editing (equal). **Chaochao Tan:** Data curation (equal); formal analysis (equal); project administration (equal); writing – original draft (equal); writing – review and editing (equal). **Xinmin Yin:** Data curation (equal); formal analysis (equal); writing – original draft (equal); writing – review and editing (equal). **Siwei Zhu:** Data curation (equal); formal analysis (equal); writing – original draft (equal); writing – review and editing (equal). **Rongyao Cai:** Data curation (equal); formal analysis (equal); writing – original draft (equal); writing – review and editing (equal). **Chunhong Liao:** Data curation (equal); formal analysis (equal); writing – original draft (equal); writing – review and editing (equal). **Yifei Wu:** Data curation (equal); formal analysis (equal); writing – original draft (equal); writing – review and editing (equal). **Qihong Zeng:** Data curation (equal); formal analysis (equal); writing – original draft (equal); writing – review and editing (equal). **Chengzhi Cai:** Data curation (equal); formal analysis (equal); writing – original draft (equal); writing – review and editing (equal). **Wang Xie:** Data curation (equal); formal analysis (equal); writing – original draft (equal); writing – review and editing (equal). **Xiangyu He:** Data curation (equal); formal analysis (equal); writing – original draft (equal); writing – review and editing (equal). **Hao‐quan Wen:** Data curation (equal); formal analysis (equal); writing – original draft (equal); writing – review and editing (equal). **Guomin Lin:** Conceptualization (equal); formal analysis (equal); writing – original draft (equal); writing – review and editing (equal). **Qingqing He:** Data curation (equal); formal analysis (equal); writing – original draft (equal); writing – review and editing (equal). **Tingting He:** Data curation (equal); formal analysis (equal); writing – original draft (equal); writing – review and editing (equal). **Peng Gu:** Project administration (equal); writing – original draft (equal); writing – review and editing (equal). **Chang‐jun Liu:** Conceptualization (lead); formal analysis (equal); project administration (lead); writing – original draft (lead); writing – review and editing (lead).

## FUNDING INFORMATION

This study was supported by Scientific Research Project of Health Commission of Hunan Province (D202304016971).

## CONFLICT OF INTEREST STATEMENT

The authors have no conflict of interest.

## INSTITUTIONAL REVIEW BOARD STATEMENT

The study was conducted in accordance with the Declaration of Helsinki, and approved by the Ethics Committee of the Hunan Provincial People's Hospital (Approval No.: Research 2022‐53).

## INFORMED CONSENT STATEMENT

Informed consent was obtained from all subjects involved in the study.

## Supporting information


Appendix S1.

## Data Availability

Data can be provided by the corresponding author upon reasonable request.

## References

[1] Chan E , Berlin J . Biliary tract cancers: understudied and poorly understood. J Clin Oncol. 2015;33(16):1845‐1848. 25918294 10.1200/JCO.2014.59.7591

[2] Lazcano‐Ponce EC , Miquel JF , Muñoz N , et al. Epidemiology and molecular pathology of gallbladder cancer. CA Cancer J Clin. 2001;51(6):349‐364. 11760569 10.3322/canjclin.51.6.349

[3] Bridgewater JA , Goodman KA , Kalyan A , Mulcahy MF . Biliary tract cancer: epidemiology, radiotherapy, and molecular profiling. Am Soc Clin Oncol Educ Book. 2016;35:e194‐e203. 27249723 10.1200/EDBK_160831

[4] Xiuying G , Zheng R , Zeng H , et al. Incidence and mortality of biliary tract cancer in cancer registration areas in China, 2008~2012. China Cancer. 2018;27:481‐488.

[5] Rizzo A , Brandi G . Adjuvant systemic treatment in resected biliary tract cancer: state of the art, controversies, and future directions. Cancer Treat Res Commun. 2021;27:100334. 33592563 10.1016/j.ctarc.2021.100334

[6] Takada T , Amano H , Yasuda H , et al. Is postoperative adjuvant chemotherapy useful for gallbladder carcinoma? A phase III multicenter prospective randomized controlled trial in patients with resected pancreaticobiliary carcinoma. Cancer. 2002;95(8):1685‐1695. 12365016 10.1002/cncr.10831

[7] Bridgewater J , Fletcher P , Palmer DH , et al. Long‐term outcomes and exploratory analyses of the randomized phase III BILCAP study. J Clin Oncol. 2022;40(18):2048‐2057. 35316080 10.1200/JCO.21.02568

[8] Primrose JN , Fox RP , Palmer DH , et al. Capecitabine compared with observation in resected biliary tract cancer (BILCAP): a randomised, controlled, multicentre, phase 3 study. Lancet Oncol. 2019;20(5):663‐673. 30922733 10.1016/S1470-2045(18)30915-X

[9] Shroff RT , Kennedy EB , Bachini M , et al. Adjuvant therapy for resected biliary tract cancer: ASCO clinical practice guideline. J Clin Oncol. 2019;37(12):1015‐1027. 30856044 10.1200/JCO.18.02178

[10] CSCO . Chinese Society of Clinical Oncology: Clinical practice guideline for biliary tract malignant tumors. People's Medical Publishing House; 2020.

[11] Edeline J , Benabdelghani M , Bertaut A , et al. Gemcitabine and oxaliplatin chemotherapy or surveillance in resected biliary tract cancer (PRODIGE 12‐ACCORD 18‐UNICANCER GI): a randomized phase III study. J Clin Oncol. 2019;37(8):658‐667. 30707660 10.1200/JCO.18.00050

[12] Nakachi K , Konishi M , Ikeda M , et al. A randomized phase III trial of adjuvant S‐1 therapy vs. observation alone in resected biliary tract cancer: Japan Clinical Oncology Group Study (JCOG1202, ASCOT). Jpn J Clin Oncol. 2018;48(4):392‐395. 29462482 10.1093/jjco/hyy004

[13] Rizzo A , Ricci AD , Brandi G . Durvalumab: an investigational anti‐PD‐L1 antibody for the treatment of biliary tract cancer. Expert Opin Investig Drugs. 2021;30(4):343‐350. 10.1080/13543784.2021.1897102 33645367

[14] Valle JW , Lamarca A , Goyal L , Barriuso J , Zhu AX . New horizons for precision medicine in biliary tract cancers. Cancer Discov. 2017;7(9):943‐962. 28818953 10.1158/2159-8290.CD-17-0245 PMC5586506

[15] Berchuck JE , Facchinetti F , DiToro DF , et al. The clinical landscape of cell‐free DNA alterations in 1671 patients with advanced biliary tract cancer. Ann Oncol. 2022;33(12):1269‐1283. 36089135 10.1016/j.annonc.2022.09.150

[16] Conci S , Ruzzenente A , Simbolo M , et al. Multigene mutational profiling of biliary tract cancer is related to the pattern of recurrence in surgically resected patients. Updates Surg. 2020;72(1):119‐128. 32020551 10.1007/s13304-020-00718-5

[17] Churi CR , Shroff R , Wang Y , et al. Mutation profiling in cholangiocarcinoma: prognostic and therapeutic implications. PLoS One. 2014;9(12):e115383. 25536104 10.1371/journal.pone.0115383 PMC4275227

[18] Nepal C , O'Rourke CJ , Oliveira DVNP , et al. Genomic perturbations reveal distinct regulatory networks in intrahepatic cholangiocarcinoma. Hepatology. 2018;68(3):949‐963. 29278425 10.1002/hep.29764 PMC6599967

[19] Boerner T , Drill E , Pak LM , et al. Genetic determinants of outcome in intrahepatic cholangiocarcinoma. Hepatology. 2021;74(3):1429‐1444. 33765338 10.1002/hep.31829 PMC8713028

[20] Jolissaint JS , Soares KC , Seier KP , et al. Intrahepatic cholangiocarcinoma with lymph node metastasis: treatment‐related outcomes and the role of tumor genomics in patient selection. Clin Cancer Res. 2021;27(14):4101‐4108. 33963001 10.1158/1078-0432.CCR-21-0412 PMC8282702

[21] Chae H , Kim D , Yoo C , et al. Therapeutic relevance of targeted sequencing in management of patients with advanced biliary tract cancer: DNA damage repair gene mutations as a predictive biomarker. Eur J Cancer. 2019;120:31‐39. 31476489 10.1016/j.ejca.2019.07.022

[22] Biliary Tract Cancers NCCN Guidelines Version 1. National Comprehensive Cancer Network; 2023.

[23] Cao J , Chen L , Li H , et al. An accurate and comprehensive clinical sequencing assay for cancer targeted and immunotherapies. Oncologist. 2019;24(12):e1294‐e1302. 31409745 10.1634/theoncologist.2019-0236 PMC6975945

[24] Sha D , Jin Z , Budczies J , Kluck K , Stenzinger A , Sinicrope FA . Tumor mutational burden as a predictive biomarker in solid tumors. Cancer Discov. 2020;10(12):1808‐1825. 33139244 10.1158/2159-8290.CD-20-0522 PMC7710563

[25] Zhang W , Shi J , Wang Y , et al. Next‐generation sequencing‐guided molecular‐targeted therapy and immunotherapy for biliary tract cancers. Cancer Immunol Immunother. 2021;70(4):1001‐1014. 33095329 10.1007/s00262-020-02745-y PMC10992508

[26] Lin J , Cao Y , Yang X , et al. Mutational spectrum and precision oncology for biliary tract carcinoma. Theranostics. 2021;11(10):4585‐4598. 33754015 10.7150/thno.56539 PMC7978308

[27] Subbiah V , Lassen U , Élez E , et al. Dabrafenib plus trametinib in patients with BRAF(V600E)‐mutated biliary tract cancer (ROAR): a phase 2, open‐label, single‐arm, multicentre basket trial. Lancet Oncol. 2020;21(9):1234‐1243. 32818466 10.1016/S1470-2045(20)30321-1

[28] Chen JS , Hsu C , Chiang NJ , et al. A KRAS mutation status‐stratified randomized phase II trial of gemcitabine and oxaliplatin alone or in combination with cetuximab in advanced biliary tract cancer. Ann Oncol. 2015;26(5):943‐949. 25632066 10.1093/annonc/mdv035

[29] Murakami Y , Uemura K , Sudo T , et al. Prognostic factors after surgical resection for intrahepatic, hilar, and distal cholangiocarcinoma. Ann Surg Oncol. 2011;18(3):651‐658. 20945107 10.1245/s10434-010-1325-4

[30] Creasy JM , Goldman DA , Dudeja V , et al. Systemic chemotherapy combined with resection for locally advanced gallbladder carcinoma: surgical and survival outcomes. J Am Coll Surg. 2017;224(5):906‐916. 28216422 10.1016/j.jamcollsurg.2016.12.058 PMC5409857

[31] Groot Koerkamp B , Wiggers JK , Gonen M , et al. Survival after resection of perihilar cholangiocarcinoma‐development and external validation of a prognostic nomogram. Ann Oncol. 2015;26(9):1930‐1935. 26133967 10.1093/annonc/mdv279 PMC4754626

