# Supplementary material for: Mutational spectrum for guiding the decision of adjuvant treatment in patients with resected biliary tract carcinoma
Source: Cancer Med. 2023 Jun 21;12(15):16076–86. doi: 10.1002/cam4.6261 (PMC10469713; doi:10.1002/cam4.6261)
Supplement: Supplementary file 1 — Appendix S1. [file CAM4-12-16076-s001.docx]

Supplementary Table 1. List of adjuvant treatment regimens received by the 35 patients

| Patient ID | Cancer subtype | Adjuvant treatment regimen | *TMB value |
| --- | --- | --- | --- |
| 1 | iCCA | Platinum-based |  |
| 2 | iCCA | S-1 based |  |
| 3 | iCCA | Targeted therapy (Apatinib) |  |
| 4 | eCCA | S-1 based |  |
| 5 | iCCA | S-1 based |  |
| 6 | iCCA | Immune checkpoint inhibitor-based | 1.6 |
| 7 | iCCA | Immune checkpoint inhibitor-based | 2.7 |
| 8 | GBC | S-1 based |  |
| 9 | iCCA | Capecitabine |  |
| 10 | eCCA | Capecitabine |  |
| 11 | iCCA | S-1 based |  |
| 12 | iCCA | S-1 based |  |
| 13 | eCCA | Capecitabine |  |
| 14 | iCCA | S-1 based |  |
| 15 | iCCA | Capecitabine |  |
| 16 | pCCA | S-1 based |  |
| 17 | iCCA | Capecitabine |  |
| 18 | iCCA | S-1 based |  |
| 19 | iCCA | S-1 based |  |
| 20 | iCCA | S-1 based |  |
| 21 | iCCA | S-1 based |  |
| 22 | pCCA | Targeted therapy |  |
| 23 | GBC | Capecitabine |  |
| 24 | GBC | Immune checkpoint inhibitor-based | 2.8 |
| 25 | iCCA | Targeted therapy (Lenvatinib) |  |
| 26 | pCCA | Targeted therapy |  |
| 27 | eCCA | S-1 based |  |
| 28 | iCCA | Capecitabine |  |
| 29 | pCCA | Platinum-based |  |
| 30 | pCCA | S-1 based |  |
| 31 | pCCA | Chemoradiotherapy |  |
| 32 | iCCA | Immune checkpoint inhibitor-based | 5.1 |
| 33 | iCCA | Targeted therapy (trametinib+dabrafenib) |  |
| 34 | iCCA | Immune checkpoint inhibitor-based | 6.2 |
| 35 | pCCA | S-1 based |  |

*Only the TMB value of patients receiving immune checkpoint inhibitor-based treatment were shown.

Supplementary Table 2. Univariate analysis of each single mutation

| Mutated gene | Log-rank P value | Selected as ‘favorable mutations’ | Selected as ‘unfavorable mutations’ |
| --- | --- | --- | --- |
| *ACVR1B* | 0.163 | Yes |  |
| *APC* | 0.243 |  |  |
| *AR* | 0.146 | Yes |  |
| *ARID1A* | 0.170 |  | Yes |
| *ARID1B* | 0.719 |  |  |
| *ARID2* | 0.604 |  |  |
| *ATM* | 0.314 |  |  |
| *ATR* | 0.719 |  |  |
| *BAP1* | 0.541 |  |  |
| *BRAF* | 0.737 |  |  |
| *BRCA2* | 0.239 |  |  |
| *CCNE1* | 0.281 |  |  |
| *CDK12* | 0.446 |  |  |
| *CDKN2A* | 0.157 |  | Yes |
| *CHD2* | 0.960 |  |  |
| *CTNNB1* | 0.019 | Yes |  |
| *EP300* | 0.617 |  |  |
| *EPHA2* | 0.796 |  |  |
| *ERBB2* | 0.273 |  |  |
| *ERBB3* | 0.069 | Yes |  |
| *ERBB4* | 0.804 |  |  |
| *FAT1* | 0.305 |  |  |
| *FAT3* | 0.613 |  |  |
| *FGF3* | 0.496 |  |  |
| *FGFR2* | 0.180 |  | Yes |
| *FRS2* | 0.667 |  |  |
| *GLI3* | 0.672 |  |  |
| *GNAS* | 0.865 |  |  |
| *KAT6A* | 0.204 |  |  |
| *KMT2C* | 0.391 |  |  |
| *KMT2D* | 0.576 |  |  |
| *KRAS* | 0.638 |  |  |
| *LRP1B* | 0.598 |  |  |
| *LRP2* | 0.096 | Yes |  |
| *MDM2* | 0.943 |  |  |
| *MUC16* | 0.296 |  |  |
| *NF1* | 0.085 |  | Yes |
| *NF2* | 0.020 |  | Yes |
| *PBRM1* | 0.161 |  | Yes |
| *PIK3CA* | 0.092 |  | Yes |
| *PRKDC* | 0.495 |  |  |
| *PTEN* | 0.379 |  |  |
| *RARA* | 0.830 |  |  |
| *RBM10* | 0.460 |  |  |
| *SF3B1* | 0.959 |  |  |
| *SMAD4* | 0.742 |  |  |
| *SPTA1* | 0.847 |  |  |
| *TERT* | 0.783 |  |  |
| *TGFBR1* | 0.097 |  | Yes |
| *TP53* | 0.880 |  |  |
| *IDH1/2* | 0.725 |  |  |
| *DDR pathway | 0.796 |  |  |
| *FGF pathway | 0.614 |  |  |

*Germline or somatic mutations in *ATM*, *ATR*, *BAP1*, and *BRCA2* were classified as DDR pathway mutations, and somatic mutations in *FGF3* and *FGFR2* were classified as fibroblast growth factor (FGF) pathway mutations.

Supplementary Figure 1. Flow chart of patients’ enrollment


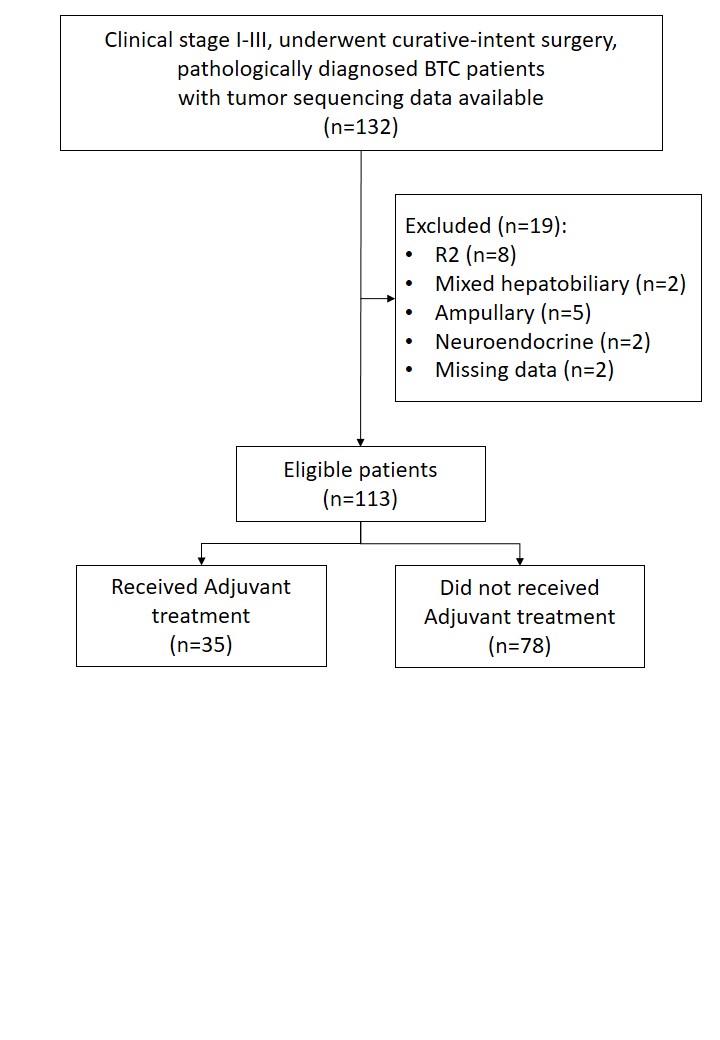


Supplementary Figure 2. Univariate analysis of the prognostic value of clinical factors


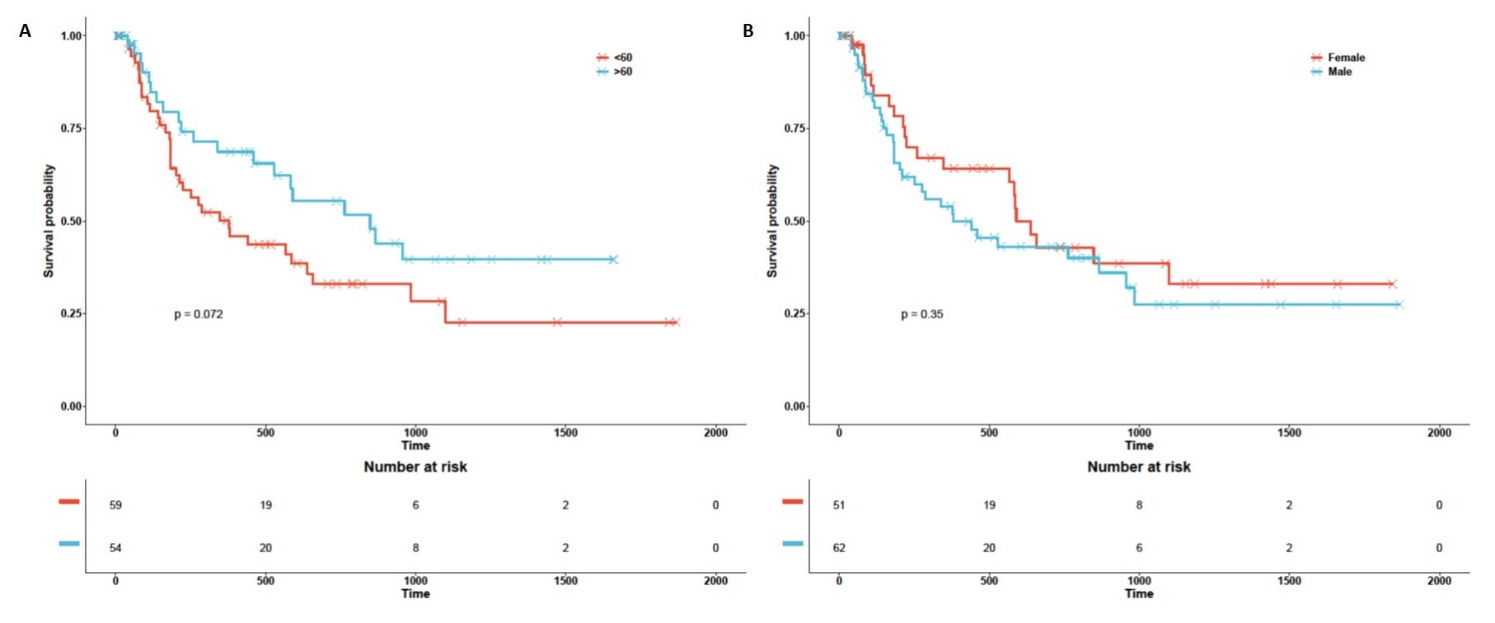

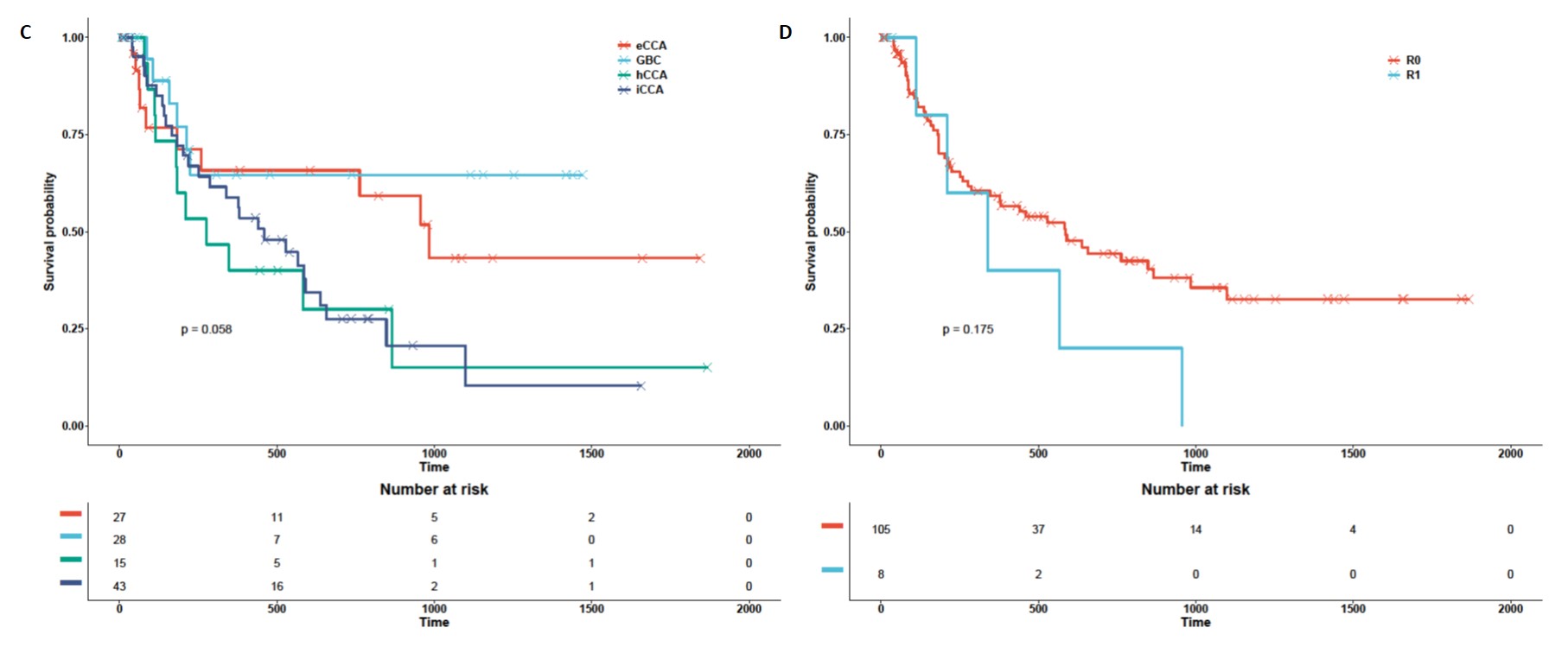

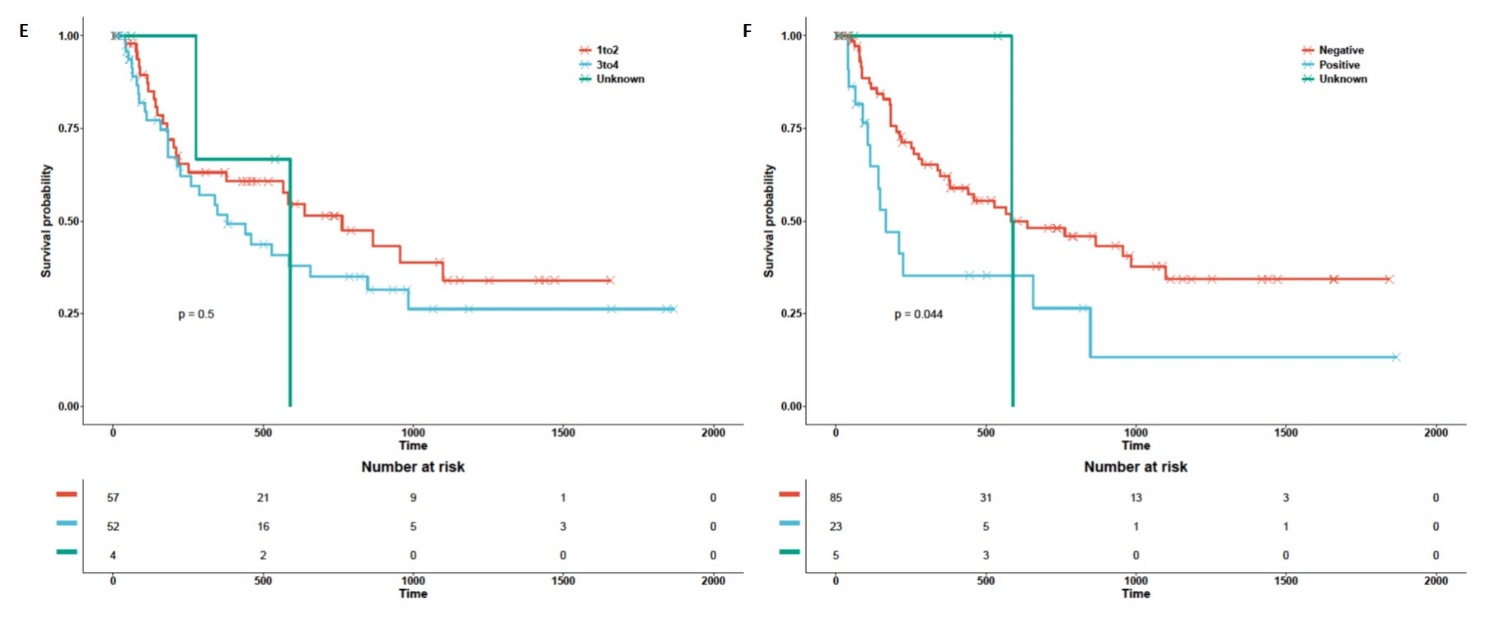

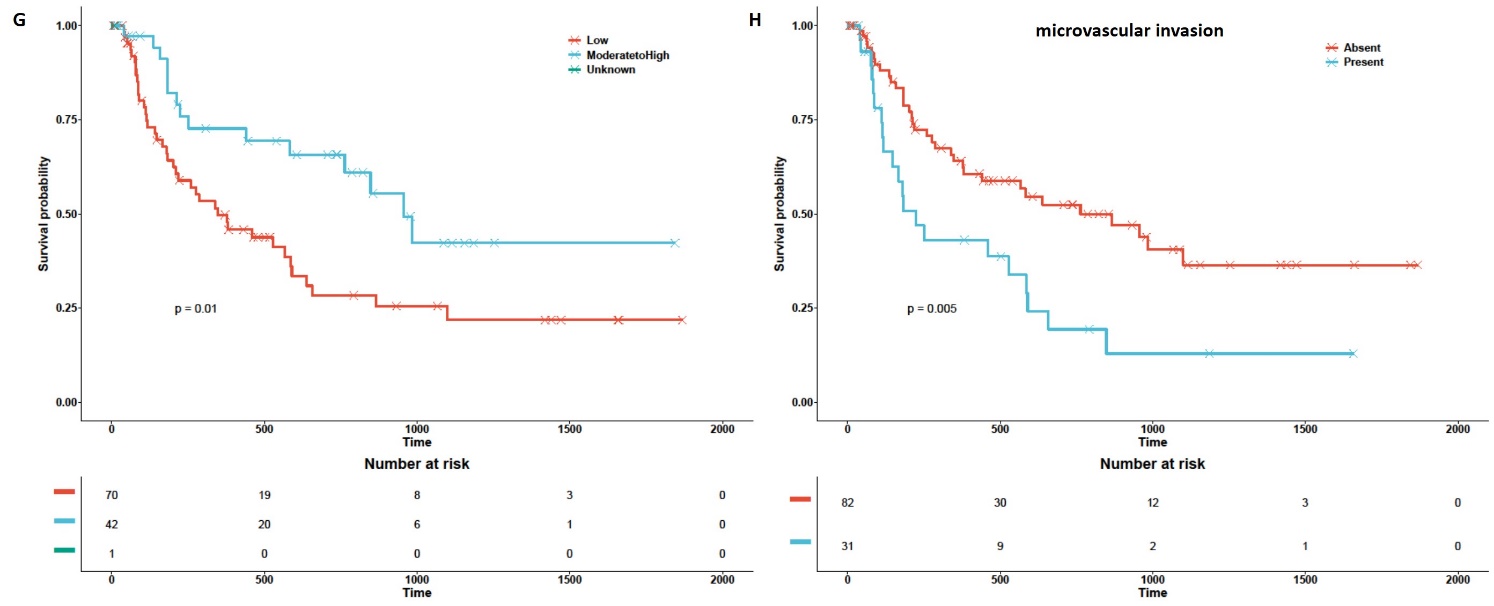

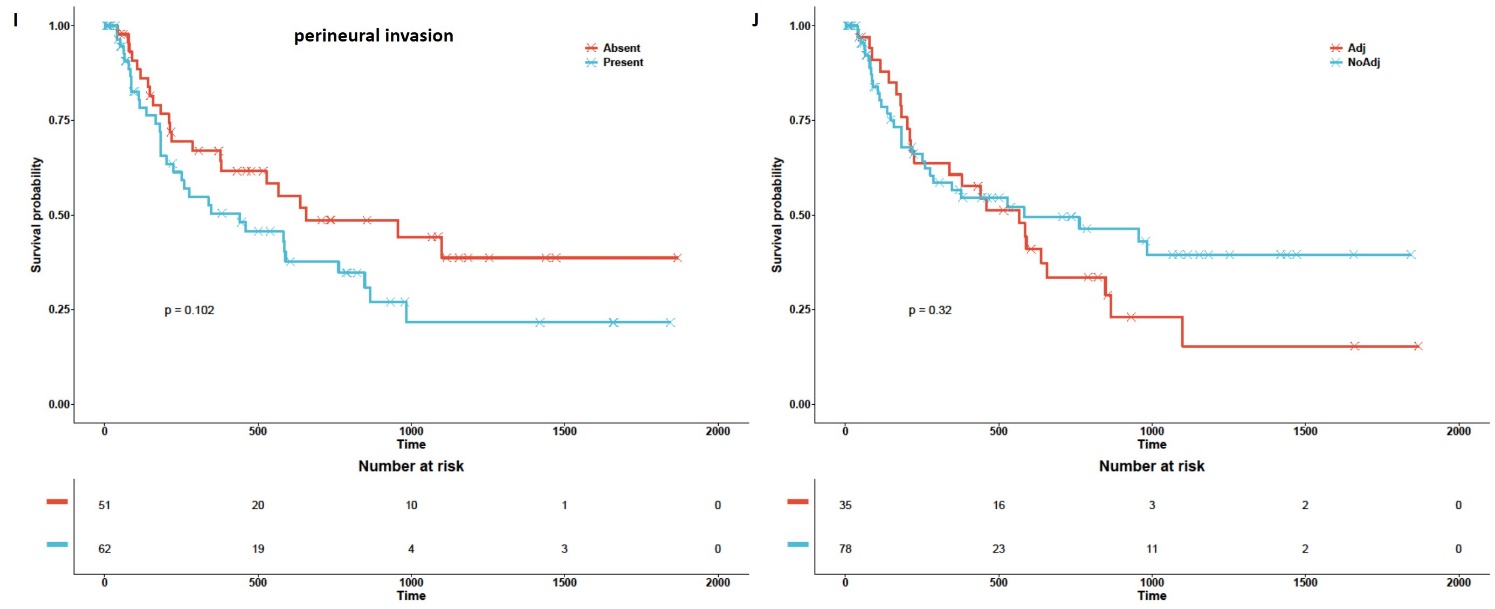


Univariate analysis of the DFS between patients with different (A) age (≤60 vs. >60); (B) sex (female vs. male); (C) Cancer type (eCCA vs. GBC vs. hCCA vs. iCCA); (D) resection margin status (R0 vs. R1); (E) T staging (1-2 vs. 3-4 vs. unknown); (F) N staging (negative vs. positive vs. unknown); (G) tumor differentiation (low vs. moderate/high vs. unknown); (H) microvascular invasion (absent vs. present); (I) perineural invasion (absent vs. present); and (J) adjuvant treatment (with vs. without).

Supplementary Figure 3. Co-occurring and mutually exclusive mutations


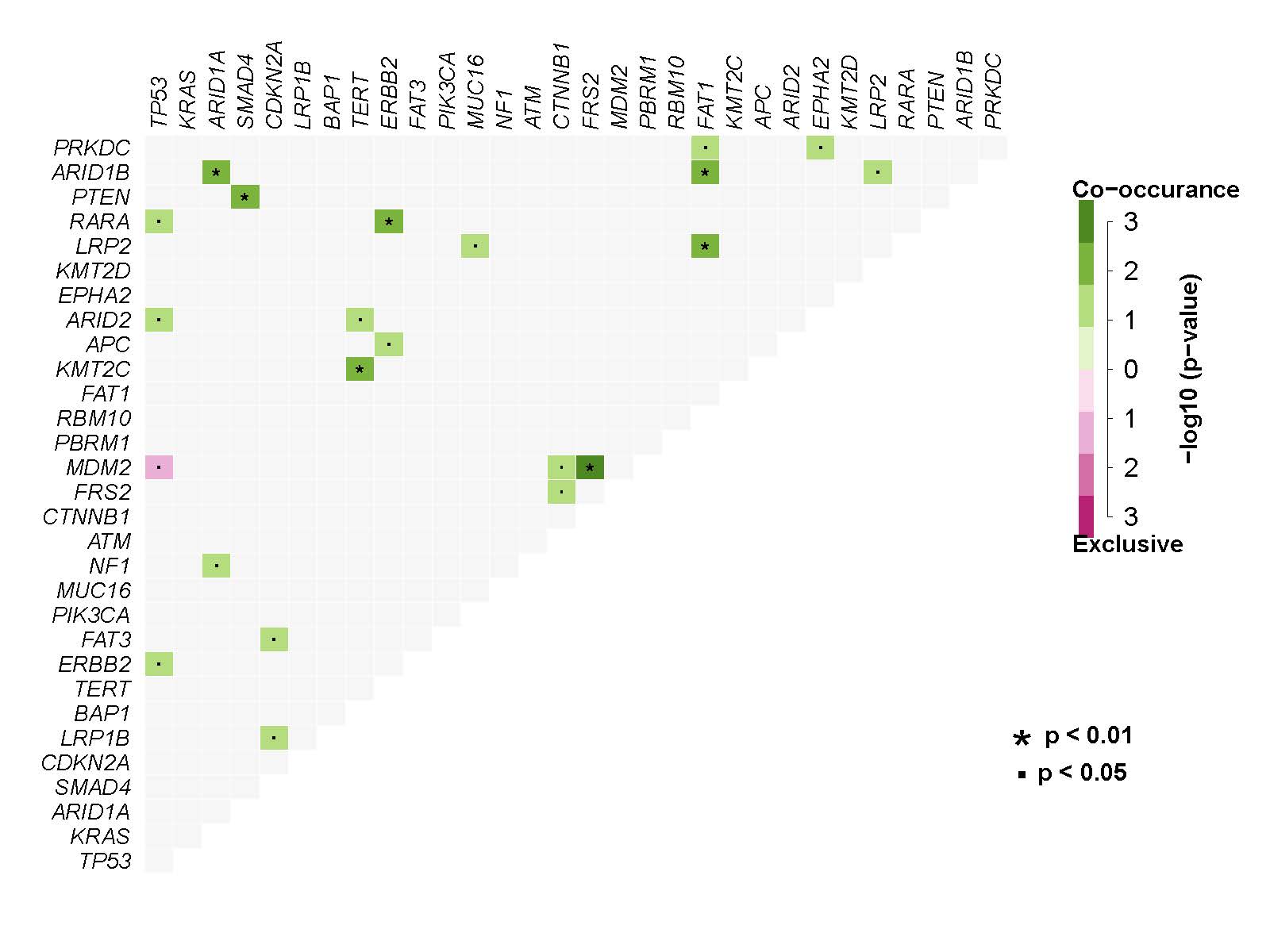


Supplementary Figure 4. Kaplan-Meier curves of the selected genes with favorable mutations


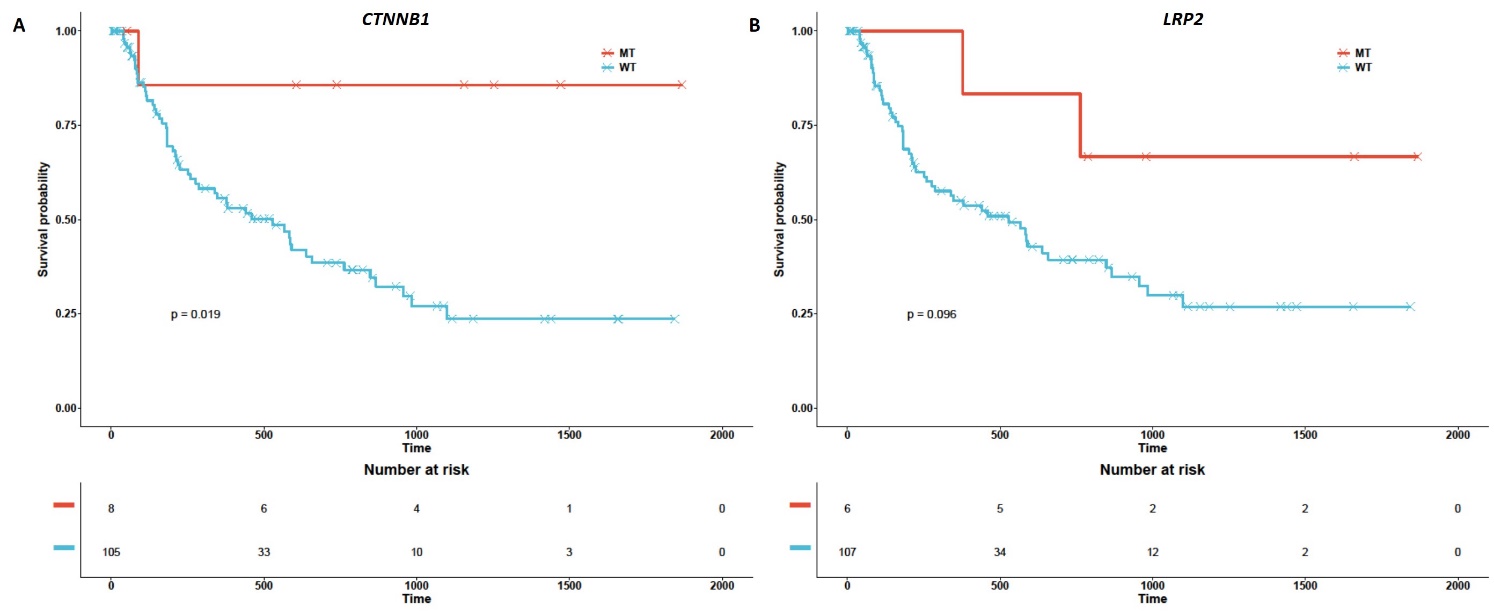

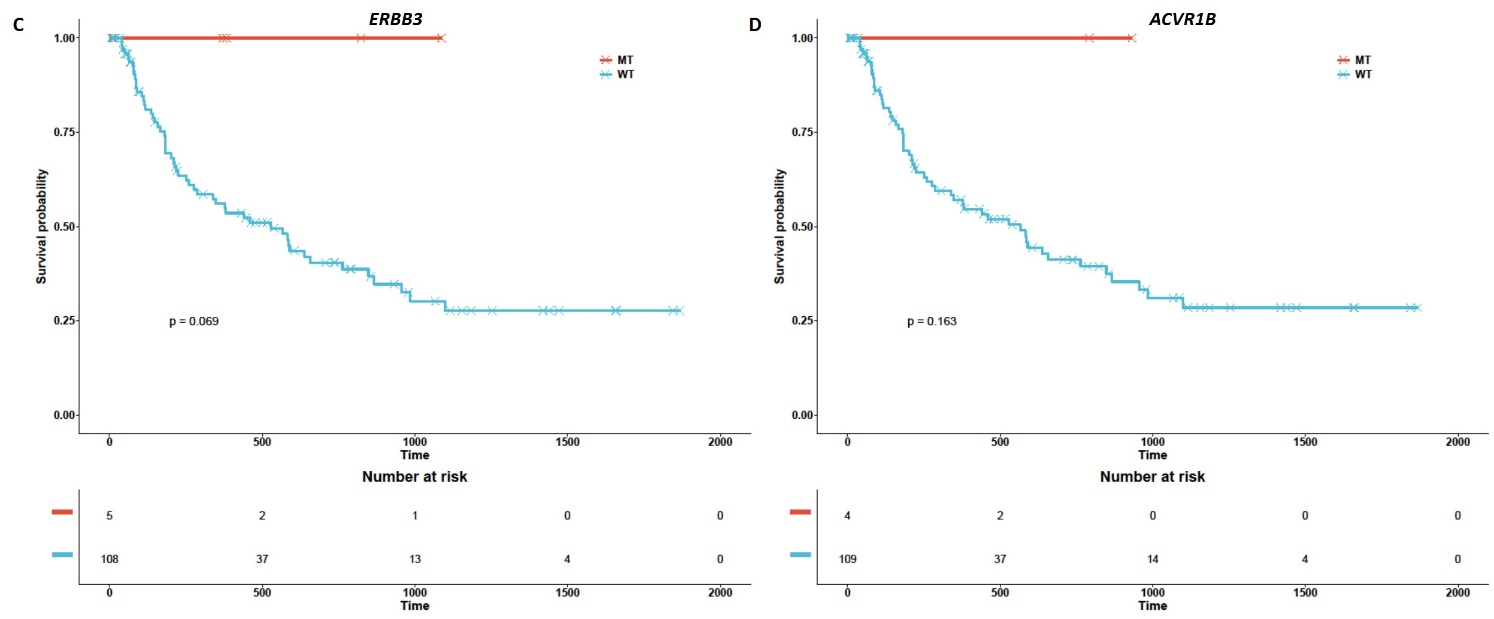


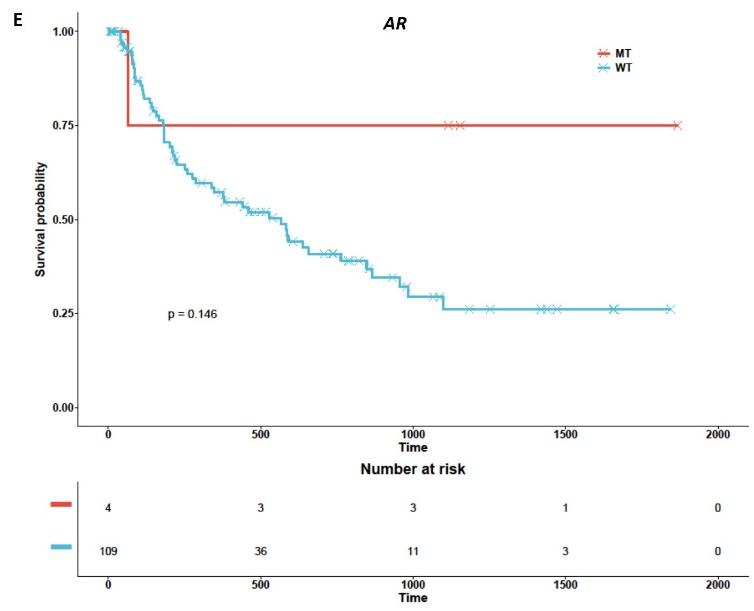


Kaplan-Meier curves comparing patients with and without mutations in (A) *CTNNB1*; (B) *LRP2*; (C) *ERBB3*; (D) *ACVR1B*; and (E) *AR*.

Supplementary Figure 5. Kaplan-Meier curves of the selected genes with unfavorable mutations


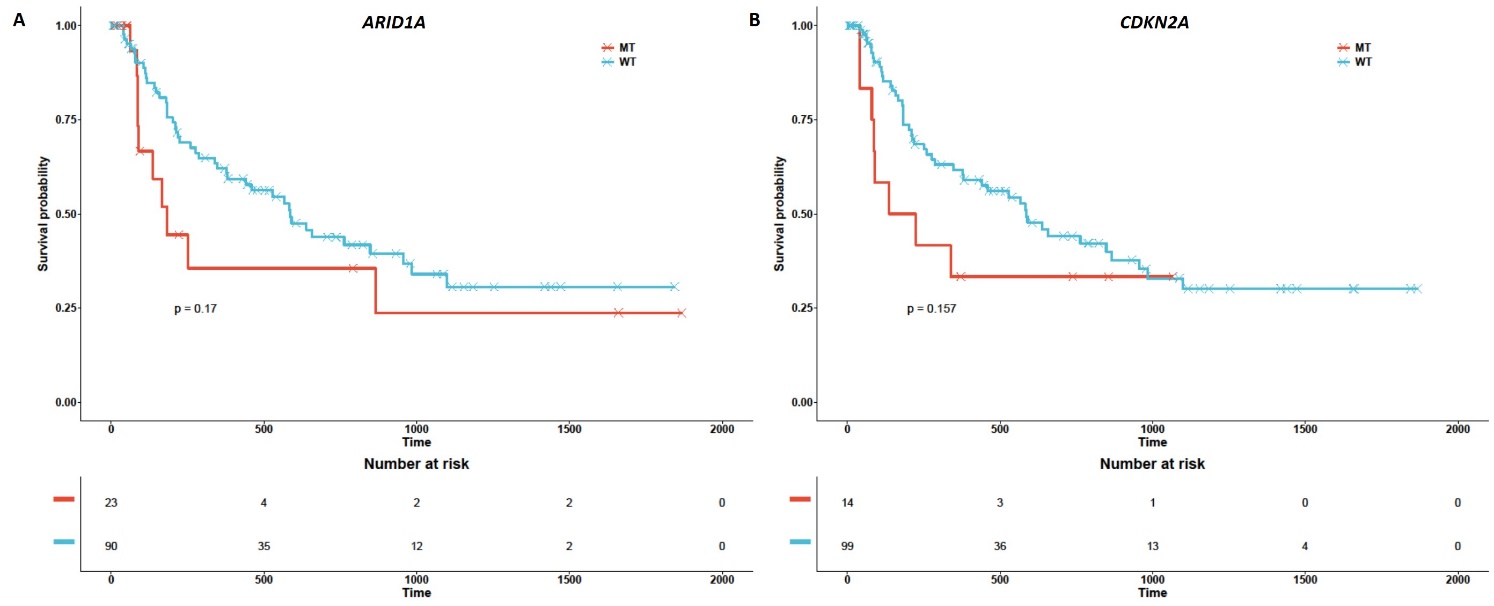

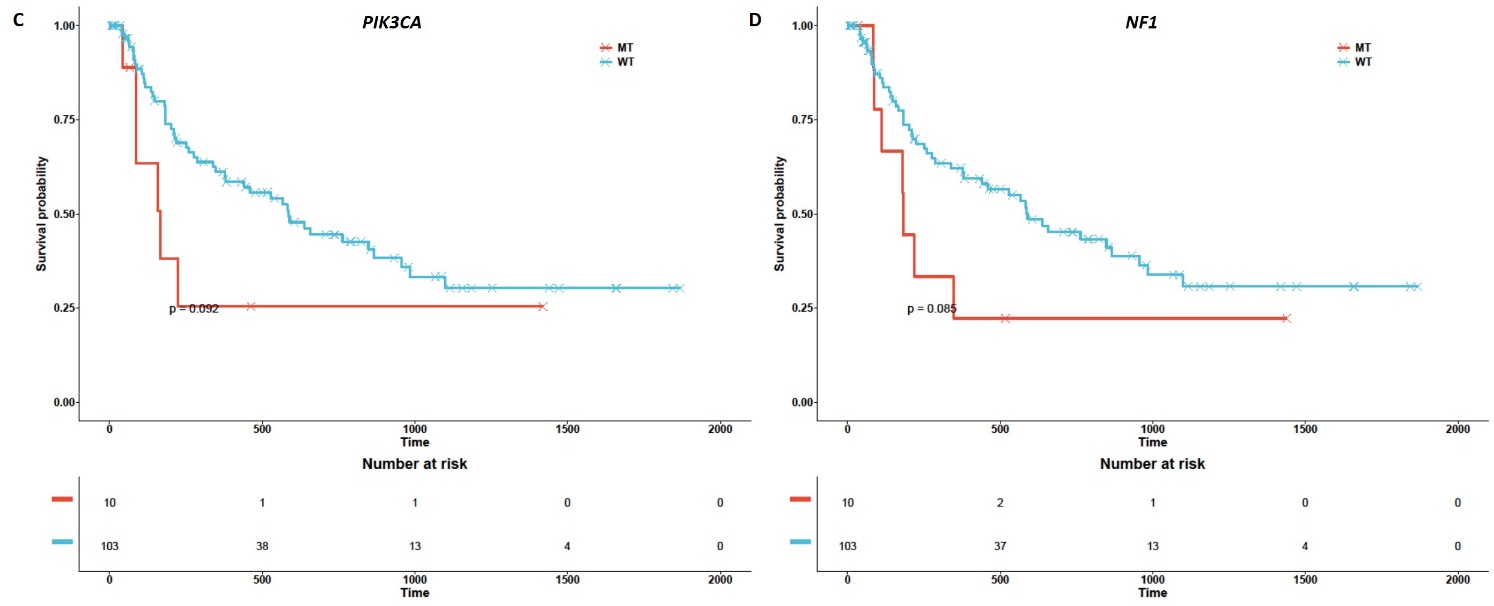

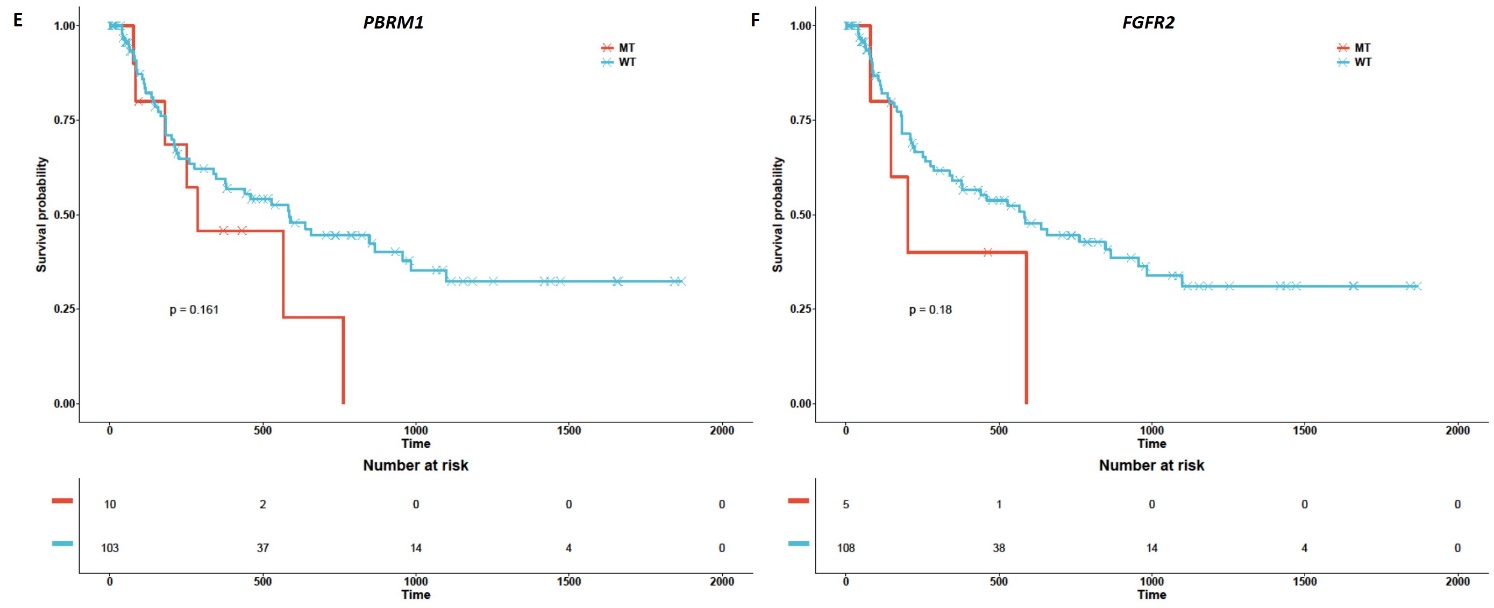

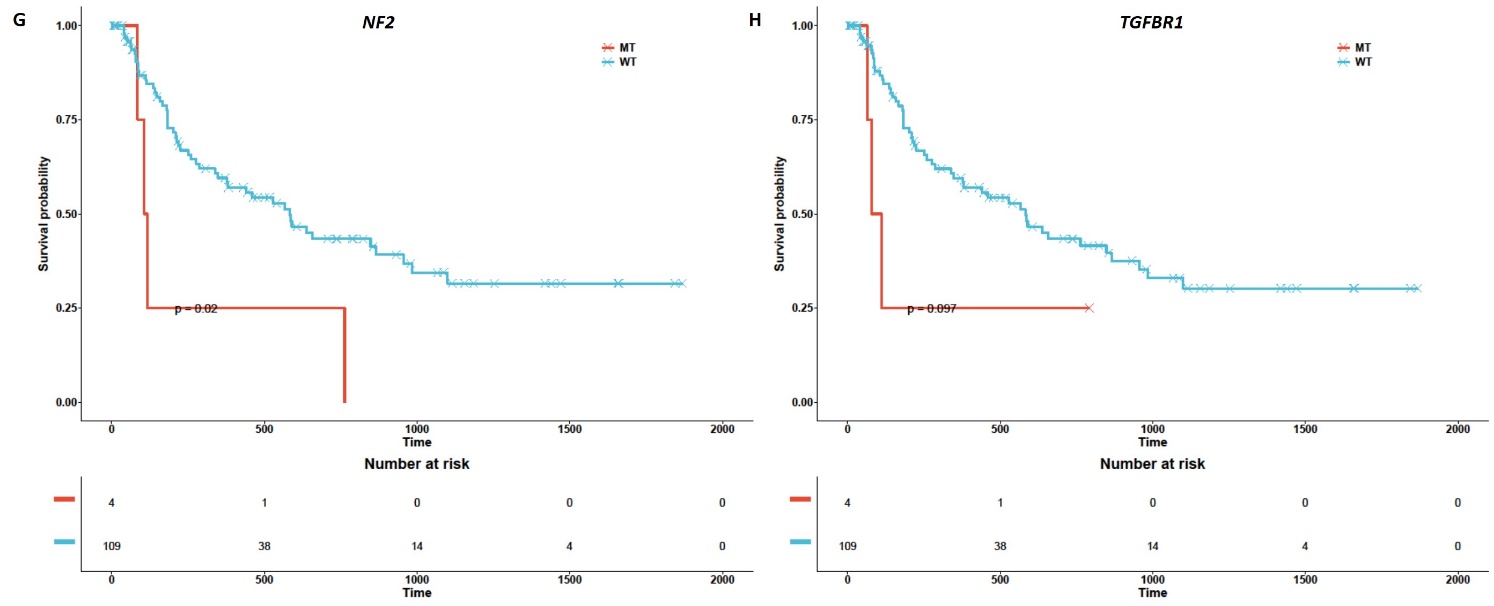


Kaplan-Meier curves comparing patients with and without mutations in (A) *ARID1A*; (B) *CDKN2A*; (C) *PIK3CA*; (D) *NF1*; (E) *PBRM1*; (F) *FGFR2*; (G) *NF2*; and (H) *TGFBR1*.
